# Approximate Invariance of Metabolic Energy per Synapse during Development in Mammalian Brains

**DOI:** 10.1371/journal.pone.0033425

**Published:** 2012-03-27

**Authors:** Jan Karbowski

**Affiliations:** Institute of Biocybernetics and Biomedical Engineering, Polish Academy of Sciences, Warsaw, Poland; University of Michigan, United States of America

## Abstract

During mammalian development the cerebral metabolic rate correlates qualitatively with synaptogenesis, and both often exhibit bimodal temporal profiles. Despite these non-monotonic dependencies, it is found based on empirical data for different mammals that regional metabolic rate per synapse is approximately conserved from birth to adulthood for a given species (with a slight deviation from this constancy for human visual and temporal cortices during adolescence). A typical synapse uses about 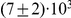 glucose molecules per second in primate cerebral cortex, and about five times of that amount in cat and rat visual cortices. A theoretical model for brain metabolic expenditure is used to estimate synaptic signaling and neural spiking activity during development. It is found that synaptic efficacy is generally inversely correlated with average firing rate, and, additionally, synapses consume a bulk of metabolic energy, roughly 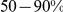 during most of the developmental process (except human temporal cortex 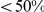). Overall, these results suggest a tight regulation of brain electrical and chemical activities during the formation and consolidation of neural connections. This presumably reflects strong energetic constraints on brain development.

## Introduction

The proper functioning of neural circuits depends on their proper wiring [1], [2], [3], [4], [5], [6], [7]. The right connectivity diagram is achieved during development that is both genetically and activity driven [8], [9], [10], and which probably has been optimized in the long evolutionary process [11], [12]. Despite the widespread application of recording, imaging and molecular techniques [13], [14], along with modeling studies [2], [15], [16], it is fair to say that our understanding of brain connectivity development is still very limited, and mostly qualitative. Nevertheless, the formation of neural circuits is an important problem in neuroscience, as its understanding may shed some light on structural memory formation in the brain and various developmental disorders [17]. Moreover, synaptic development like every physical process requires some energy. A natural question is how much does it cost, and whether this cost changes during development. It is known that information processing in the brain is metabolically expensive [18], [19], [20]. Specifically, energy consumption in mammalian brains increases fast with brain size, far more than in the rest of the body [21].

The process of synaptogenesis, i.e. formation of synaptic connections, can be region specific and can have a complicated time-course, often bimodal with synaptic overproduction early in the development [22], [23], [24], [25], [26], [27], [28], [29]. However, we do not know whether and how this process correlates with the activities of participating neurons. It is also unclear, to what extent the synaptogenesis is regulated metabolically, although some qualitative correlation between the two has been noted based on their temporal characteristics [29], [30].

A couple of theoretical investigations estimated that synapses in the adult brain consume a significant portion of the overall metabolic rate [31], [32]. However, in fact, cerebral metabolic rate CMR (glucose consumption rate) depends both on neural electric discharges and on synaptic signaling, and their relative contribution is strongly controlled by a neurotransmitter release probability and synaptic density [33]. For instance, a high release probability can make synapses the major consumer of energy, and conversely, a low probability can cause action potentials to be metabolically dominant. Thus, simultaneous analysis of the cerebral metabolic rate and synaptic density during development can provide a useful quantitative information about the relative importance of these two factors. Additionally, it can yield a relationship between synaptic signaling and neural firing rates.

The main aim of this study is to address these questions in two steps. First, by collecting and analyzing empirical data on brain metabolism and synaptic density during development for different mammals. Second, by combining these data with a theoretical model for brain metabolic rate [33], in order to obtain quantitative results on the relationship energy vs. synapses. In particular, we want to establish how common across mammals are mechanisms that relate synaptogenesis with neural activities and cerebral metabolism. A secondary goal is to test the analytic model of brain metabolism against the data, which is a little extended here from its original formulation in [33]. In this model, cerebral metabolic rate is expressed solely by neural and synaptic physiological parameters that are either known or can be easily measured.

## Results

### Constancy of metabolic energy per synapse during development

**Figure 1:**
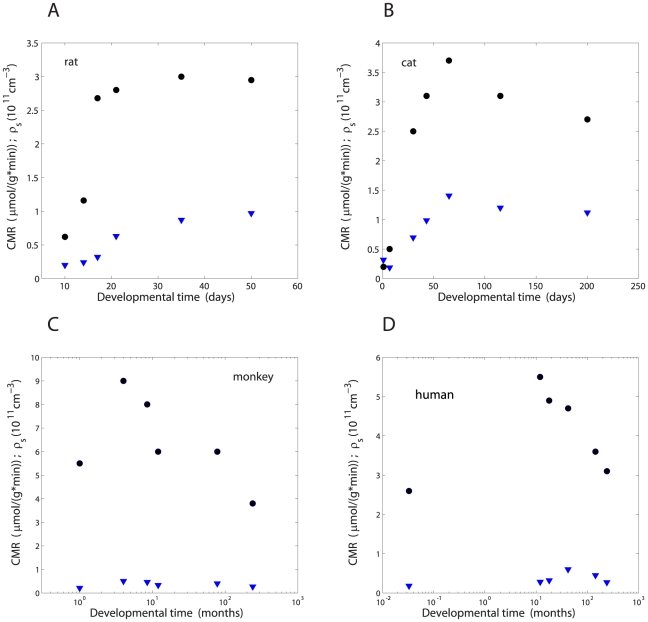
Dependence of glucose cerebral metabolic rate CMR and synaptic density 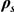 on developmental time in visual cortex of various mammals. (A) Rat; (B) Cat; (C) Monkey; (D) Human. Circles correspond to the synaptic density and triangles to CMR.

**Figure 2:**
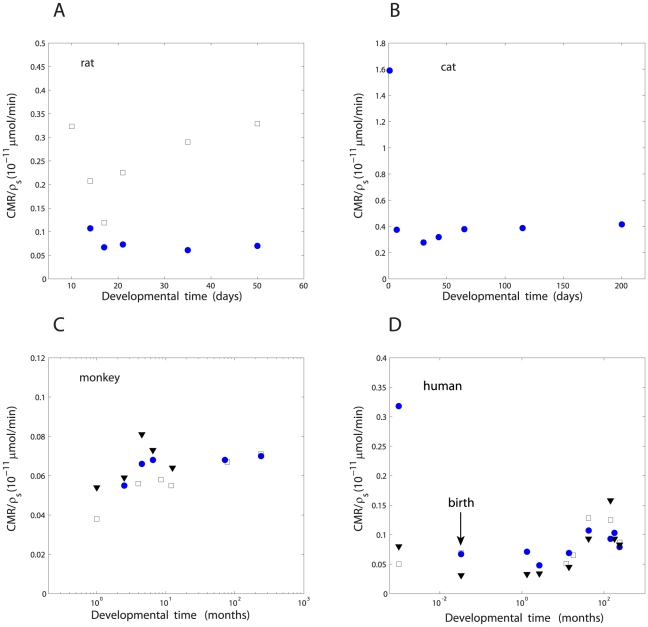
Approximate invariance of glucose cerebral metabolic rate per synapse during development. The linear fits to the data points are given in the brackets below. (A) Rat (circles - parietal cortex: 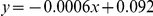, 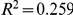, 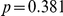; squares - visual cortex: 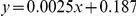, 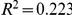, 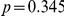). (B) Cat visual cortex (with the data point at 1 day: 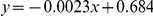, 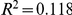, 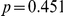; without the data point at 1 day: 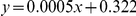, 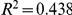, 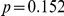). (C) Monkey (circles - frontal cortex: 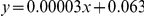, 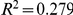, 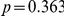; squares - visual cortex: 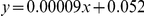, 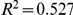, 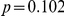; triangles - sensorimotor cortex: 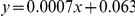, 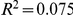, 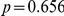). (D) Human (circles - frontal cortex: 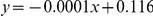, 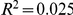, 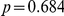; squares - visual cortex: 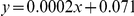, 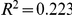, 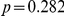; triangles - temporal cortex: 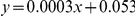, 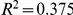, 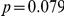). In the above fits 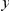 refers to CMR/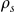 (in 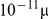
mol/min) and 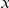 to the developmental time (either in days for rat and cat or in months for monkey and human). Note that for all fits the linear coefficient is close to zero.

Empirical data (Tables 1–3) were used to analyze the time course of synaptic density (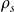) and glucose cerebral metabolic rate (CMR) during development for different mammals and brain regions (Fig. 1). For most regions both of these quantities depend non-monotonically on time, initially increasing, then reaching a maximum, and finally decreasing to adult values. In some cases, this temporal dependence is even more irregular, with more than one maximum (e.g. rhesus monkey frontal cortex and human temporal cortex for synaptic density). Overall, CMR and 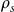 can change several-fold during development. The most extreme change is in the cat visual cortex, where 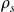 and CMR can increase by a factor of 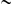
18 and 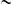
4, respectively (Table 1). However, despite these complex dependencies and variability the amount of metabolic energy per synapse, i.e. the ratio CMR/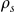, is nearly independent of the developmental time for a given species and brain area (Fig. 2; Tables 1–3). In all examined mammals and cortical regions, the quantity CMR/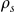 correlates weakly with the developmental time, and the linear slope in this dependence is close to zero. Moreover, these weak correlations are not statistically significant (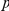 value varies from 0.08 to 0.68; Fig. 2).

**Table 1:** Synaptic and metabolic development for rat and cat cerebral cortex.

Species/region	developmental	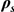	CMR	CMR/ 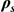	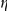
	time	[ 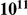 cm 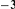 ]	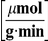	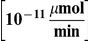	
Rat:					
 parietal cortex	14 day	2.8 [22]	0.30 [34]	0.107	0.52
	17 day	6.3 [22]	0.42 [34]	0.067	0.84
	21 day	9.0 [22]	0.66 [34]	0.073	0.77
	35 day	14.0 [22]	0.85 [34]	0.061	0.92
	adult	13.5 [22]	0.94 [34]	0.070	0.81
Rat:					
 visual cortex	10 day	0.62 [23]	0.20 [34]	0.323	0.10
	14 day	1.16 [23]	0.24 [34]	0.207	0.29
	17 day	2.68 [23]	0.32 [34]	0.119	1.19
	21 day	2.80 [23]	0.63 [34]	0.225	0.66
	35 day	3.00 [23]	0.87 [34]	0.290	0.55
	adult	2.95 [23]	0.97 [34]	0.329	0.48
Cat:					
 visual cortex	1 day	0.20 [24]	0.318 [35]	1.590	0.08
	7 day	0.50 [24]	0.187 [35]	0.374	0.42
	30 day (est)	2.50 [24]	0.696 [35]	0.278	0.89
	40–45 day	3.10 [24]	0.987 [35]	0.318	0.83
	60–70 day	3.70 [24]	1.406 [35]	0.380	0.73
	110–120 day	3.10 [24]	1.201 [35]	0.387	0.68
	adult	2.70 [24]	1.120 [35]	0.415	0.61

Developmental time refers to postnatal time. References in the brackets. Synaptic contribution 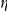 to CMR is computed from Eq. (2).

**Table 2 pone-0033425-t002:** Synaptic and metabolic development for monkey cerebral cortex.

Species/region	developmental	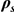	CMR	CMR/ 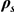	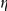
	time	[ 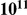 cm 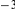 ]	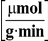	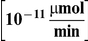	
Monkey:					
 frontal cortex	2–3 month	6.0 [26]	0.33 [36]	0.055	0.63
	4–5 month	6.1 [26]	0.40 [36]	0.066	0.53
	6–7 month	5.7 [26]	0.39 [36]	0.068	0.49
	6 year	5.0 [26]	0.34 [38]	0.068	0.47
	20 y (adult)	3.16 [26]	0.22 [38]	0.070	0.36
Monkey:					
 visual cortex	0–2 month	5.5 [25]	0.21 [37]	0.038	1.08
	2–6 month	9.0 [25]	0.50 [37]	0.056	0.94
	8–9 month	8.0 [25]	0.46 [36]	0.058	0.86
	12 month	6.0 [25]	0.33 [36]	0.055	0.78
	6–7 year	6.0 [25]	0.40 [38]	0.067	0.65
	20 y (adult)	3.8 [25]	0.27 [38]	0.071	0.49
Monkey:					
 sensorimotor crtx	0–2 month	4.78 [27], [28]	0.26 [37]	0.054	1.20
	2–3 month	5.75 [27], [28]	0.34 [36]	0.059	1.11
	4–5 month	5.44 [27], [28]	0.44 [36]	0.081	0.81
	6–7 month	5.19 [27], [28]	0.38 [36]	0.073	0.89
	12–13 month	5.78 [27], [28]	0.37 [36]	0.064	1.03

Developmental time refers to postnatal time. References in the brackets. Synaptic densities for sensorimotor cortex are arithmetic means of values in motor and somatosensory cortices.

**Table 3:** Synaptic and metabolic development for human cerebral cortex.

Species/region	developmental	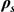	CMR	CMR/ 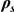	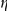
	time	[ 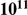 cm 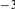 ]	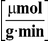	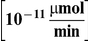	
Human:					
 frontal cortex	- (10-8) wbb(*)	0.22 [29]	0.07 [39]	0.318	0.005
	1 day	1.95 [29]	0.13 [39], [30]	0.067	0.33
	40 day	1.12 [29]	0.08 [39]	0.071	0.16
	80–83 day	3.10 [29]	0.15 [39]	0.048	0.81
	1.17 year	3.79 [29]	0.26 [30]	0.069	0.74
	3.5 year	5.24 [29]	0.56 [30]	0.107	0.70
	12 year	4.69 [29]	0.44 [30]	0.093	0.70
	15 year	4.00 [29]	0.41 [30]	0.103	0.53
	adult	3.40 [29]	0.27 [30]	0.079	0.56
Human:					
 visual cortex	- (10-8) wbb(*)	1.2 [29]	0.06 [39]	0.050	0.98
	1 day	2.6 [29]	0.18 [30]	0.069	0.71
	1 year	5.5 [29]	0.28 [30]	0.051	0.96
	1.5 year	4.9 [29]	0.32 [30]	0.065	0.75
	3.5 year	4.7 [29]	0.60 [30]	0.128	0.38
	12 year	3.6 [29]	0.45 [30]	0.125	0.39
	adult	3.1 [29]	0.27 [30]	0.087	0.56
Human:					
 temporal cortex	- (10-8) wbb(*)	0.75 [29]	0.06 [39]	0.080	0.06
	1 day	2.94 [29]	0.09 [39]	0.031	0.41
	40 day	2.10 [29]	0.07 [39]	0.033	0.30
	80–83 day	4.70 [29]	0.16 [39]	0.034	0.51
	1.17 year	5.30 [29]	0.24 [30]	0.045	0.42
	3.5 year	5.57 [29]	0.52 [30]	0.093	0.21
	12 year	2.47 [29]	0.39 [30]	0.158	0.07
	15 year	3.89 [29]	0.36 [30]	0.093	0.17
	adult	2.90 [29]	0.24 [30]	0.083	0.15

(*) Negative value refers to the weeks before birth (wbb). Positive developmental times refer to postnatal time. References in the brackets.

On average, rat brain consumes about 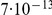 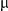
mol of glucose per minute per synapse in the parietal cortex, and 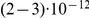 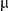
mol/min in the visual cortex (Table 1). The latter value is similar to the glucose use per synapse in the cat visual cortex (Table 1). In rhesus monkey and human cerebral cortices, there are approximately the same average baseline glucose consumptions per synapse, 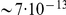 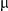
mol/min (Tables 2 and 3). From these results it follows that glucose use per synapse is smaller in large primate brains than it is in relatively small rodent of feline brains, and the difference could be five- or six-fold.

The biggest deviations from a baseline value of CMR/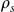 are for the human visual and temporal cortices between postnatal ages 3.5 and 12–15 years, and can be 2–3 folds above that baseline (Table 3). These numbers, however, do not seem to be relatively large, considering that CMR in that period can increase by a factor of 4–9 in relation to the minimal CMR. Nevertheless, the “energy per synapse” distinction for the (pre- and) adolescent human brain is noticeable and could suggest a different distribution of energy in the developing human neural circuits in that period in comparison to other mammals.

### Correlation between cerebral metabolic rate and synaptic density

**Figure 3:**
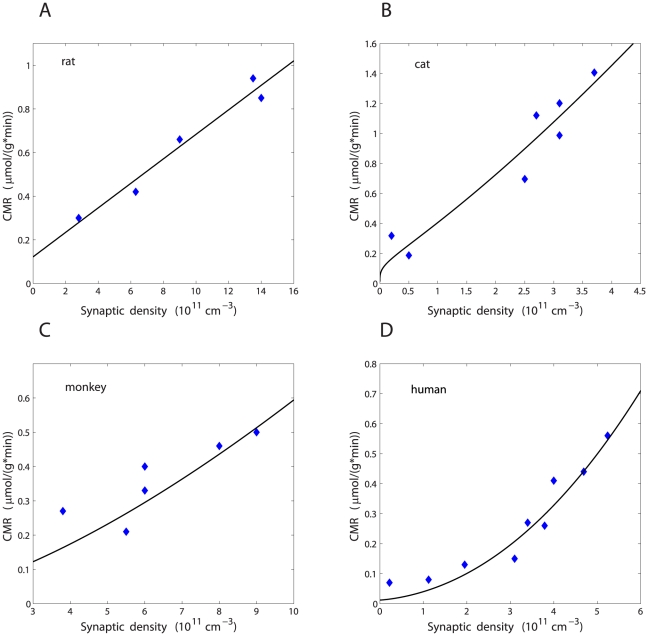
Empirical dependence of cerebral metabolic rate CMR on synaptic density 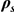 together with fits to the theoretical metabolic model. (A) Rat, parietal cortex. (B) Cat, visual cortex. (C) Monkey, visual cortex. (D) Human, frontal cortex. Empirical data are represented by diamonds, and theoretical fits by solid lines. The fitting parameters are shown in Table 4.

Empirical data on CMR and 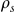 were used to find their mutual relationship (Fig. 3). This relationship is in general monotonic with high positive correlations, and can be fitted by the formula, which was derived in the Materials and Methods:
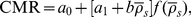
(1)where 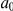 and 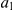 are numerical coefficients that depend on neurophysiological parameters (they are known and determined in the Materials and Methods), 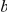 is the parameter related to synaptic signaling, 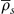 is the amplitude of synaptic density, i.e. 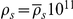 [cm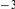]. The function 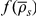 is the population average neural firing rate that changes during development with synaptic density as 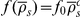. Values of the parameters 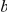, 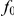, and 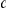 are determined by a fitting procedure to the data, and they are presented in Table 4.

**Table 4:** Best fits to the data for parameters in the relation CMR vs. 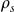 across mammals.

Species/region	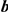 ( 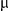 mol  s/min)	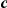	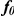 (Hz)	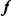 (Hz)	R 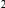	SSE
Rat: parietal cortex	0.066	0.0	0.85	0.85	0.961	0.012
Rat: visual cortex	0.071	1.02	0.73	0.4–2.2	0.674	0.181
Cat: visual cortex	0.121	0.29	1.57	1.0–2.3	0.905	0.121
Monkey: frontal cortex	0.024	0.52	0.57	1.0–1.5	0.776	0.011
Monkey: visual cortex	0.228	0.48	0.08	0.15–0.23	0.908	0.005
Monkey: sensorimotor crtx	0.692	0.03	0.09	0.1	0.262	0.013
Human: frontal cortex	0.070	1.23	0.14	0.02–1.1	0.928	0.018
Human: visual cortex	0.038	0.0	1.29	1.3	0.105	0.127
Human: temporal cortex	0.010	0.69	0.60	0.5–2.0	0.347	0.142

Generally, estimated average firing rates are rather small for all examined mammals, and on average about 1 Hz (Table 4). The smallest values are for the monkey visual and sensorimotor cortices, and the largest for the cat visual cortex. The character of the relationship between population firing rate 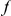 and synaptic density 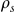 is not universal, but depends on a particular species and cortical region (Table 4). For some regions, the best fit is obtained for 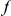 independent of 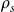 (i.e. with 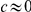). For others, we find an increase of 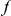 with increasing 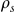, either sublinearly (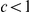) or approximately linearly (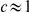). The nature of this dependence has also its influence on the relationship CMR vs. 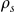. When 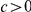, that is, when 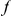 increases with 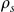, we find that CMR increases with 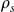 in a non-linear manner (Fig. 3B,C,D), whereas when 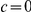, then CMR grows linearly with 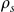 (Fig. 3A). Thus, we conclude that the dependence CMR on 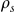 is also non-universal.

### Synaptic contribution to the cerebral metabolic rate during development

Having determined the parameters 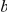, 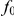, and 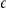, we can find a fraction of metabolic energy consumed by synaptic signaling during the development process. The fraction 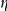 of the cerebral metabolic rate CMR taken by synapses is defined as 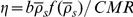, or equivalently
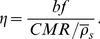
(2)


The latter expression implies that 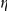 is inversely related to the metabolic energy per synapse. Indeed, although 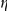 changes during the development much more than CMR/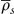 (Tables 1–3), both of these variables are negatively correlated (Table 5). The greater variability of 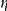 than CMR/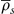 can be explained by its additional dependence on firing rate 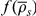, which in itself is proportional to a variable synaptic density.

**Table 5:** Correlation between metabolic energy per synapse (CMR/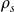) and synaptic fraction of metabolism (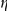).

Species/region	correlation	significance
	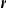	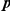
Rat: parietal cortex	−0.992	0.001
Rat: visual cortex	−0.753	0.084
Cat: visual cortex	−0.869	0.011
Monkey: frontal cortex	−0.889	0.044
Monkey: visual cortex	−0.927	0.008
Monkey: sensorimotor crtx	−0.995	0.000
Human: frontal cortex	−0.642 (0.049)	0.063 (0.908)
Human: visual cortex	−0.968 (−0.968)	0.000 (0.002)
Human: temporal cortex	−0.659 (−0.872)	0.054 (0.005)

Values in the brackets refer to 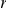 and 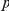 without the prenatal data points.

In general, 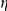 is rather high, mostly in the range 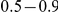 (Tables 1–3; some 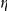 is a little above unity, which is an artifact caused by systematic errors in the fitting procedure that determines 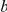, 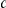, and 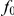). A significant exception is human temporal cortex in which synapses use for the most time considerably less than 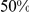 of cortical CMR. At the top of the synaptogenesis, when synaptic density is maximal, 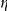 is usually very large and often around 0.8–0.9, which is greater than for the adult, but the difference is mild. From all examined mammals and cortical regions, synapses in the monkey visual and sensorimotor cortices, as well as synapses in the rat parietal cortex seem to be the most “energetic”, since they frequently use approximately 90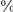 of the total cerebral glucose rate.

Overall, these results strongly suggest that excitatory synaptic signaling uses a majority of metabolic energy allocated to neurons, even at adulthood. The spiking neural activity and maintenance of negative membrane potential utilize generally far less energy, together approximately 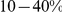, depending on the species, brain region, and developmental period.

### Relationship between synaptic efficacy and average firing rate across mammals

**Figure 4:**
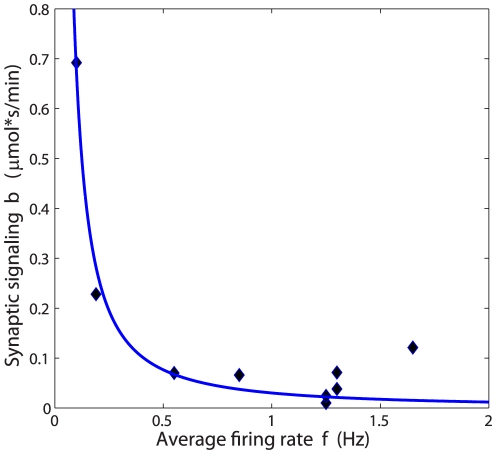
Inverse relationship between synaptic signaling and average firing rate across mammals. Values of the synaptic efficacy 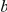 and firing rates 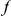 (arithmetic means) were found by fitting experimental data to the theoretical model (Table 4). Note that all data points (diamonds) coming from different species and cortical regions align into a universal curve of the form: 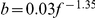 (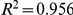, 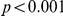).

The parameter 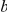 in Eq. (1) is proportional to the excitatory synaptic efficacy (or signaling; see Materials and Methods). For a given species, we can associate this parameter with the average firing rate 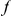, both of which were determined by fitting the theoretical model (Eq. 1) to the data (Table 4). We find that 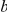 and 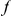 are inversely correlated across all examined cerebral regions and animals, and can be fitted quite well by a universal curve of the form (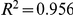; Fig. 4):
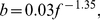
(3)where 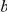 is expressed in 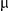
mol

sec/min. This relationship indicates that average synaptic efficacy is dependent on network spiking activity, and the higher that activity the smaller synaptic signaling. For example, for 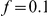 Hz we have 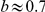, while for 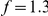 Hz we obtain 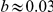, i.e. more than twenty-fold reduced synaptic efficacy. This implies that synaptic transmission is very sensitive on the average firing rate in the network, which can have functional consequences (see Discussion).

### Estimation of neurotransmitter release probability by combining data and metabolic model

Experimental data show that the probability of neurotransmitter release is the least stable parameter among synaptic parameters, and can change during the development by at least an order of magnitude [40], [41]. To test our metabolic model (see Materials and Methods), the release probability is estimated below for adult rat and cat visual cortices. In this respect, we equate the empirical value of the parameter 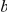 in Table 4 with the analytical formula for 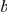 given by Eq. (15), which allows us to determine the release probability 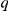. We assume that 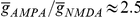, in agreement with the empirical data for adult primate brain [42]. We take the peak AMPA synaptic conductances and their decay time constants as: 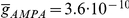 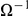 and 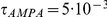 s for rat, and 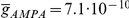 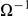 and 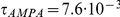 s for cat [43]. Additionally, the NMDA synaptic conductance decay time constant 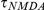 is taken as 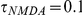 s for both species, as a standard NMDA decay time [44]. We find that the neurotransmitter release probability 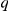 is 0.45 for adult rat visual cortex, and 0.31 for adult cat visual cortex. These values are in the range of values reported experimentally [40], [45], [46], and suggest that the metabolic model presented and used in this paper (Materials and Methods) is reliable and has a predictive power.

## Discussion

This study shows that despite temporal changes in cerebral metabolic rate CMR and synaptic density 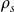 during development, often exhibiting bimodal shape, the amount of metabolic energy per synapse (CMR/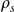) is almost invariant in the process for a given mammal and brain region (Fig. 2; Tables 1–3). This approximate constancy is even more pronounced if we take into account that many other neuroanatomical parameters, such as neuron number, dendritic tree length, and brain volume, all change non-monotonically with an animal age [47], [48], [49]. In contrast to CMR/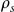, the fraction of CMR consumed by synapses, i.e. 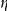, is much more variable during the development (Tables 1–3). Moreover, these two quantities are strongly negatively correlated (Table 5). For the most developmental time and cortical regions 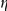 is greater than 0.5, implying that synapses use the majority of cortical metabolic energy, often close to 90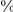 or more (Tables 1–3).

The case with the human brain is more subtle, as its visual and temporal cortices exhibit a noticeable deviation from the CMR/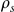 constancy during early and middle adolescence (by a factor of 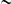
2; Table 3). In addition, 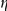 for human temporal cortex is considerably smaller than 0.5 for the most time. The increase in CMR/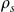 for the above regions during adolescence is associated with a simultaneous decrease in 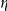, which suggests that non-synaptic part of CMR dominates over the synaptic part in this period (Table 3). It is interesting to note that the maxima of CMR/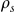 for human visual and temporal cortices between 3.5 and 12 years coincide with maxima observed in cortical volume, thickness, and surface area during the same time [50], [51], [52]. This positive (negative) correlation between CMR/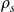 (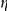) and structural cortical growth can be an indication that the latter process requires an additional energy above some baseline, which is partly generated by shunting it from the synapses.

On average, a synapse in the primate cerebral cortex consumes about 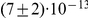 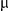
mol of glucose per minute. In rat and cat visual cortices corresponding numbers are about 5 times larger, which qualitatively agrees with a previous rough estimate that in larger brains energy per synapse should be smaller than in smaller brains [21]. These numbers translate into 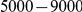 of consumed glucose molecules and 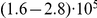 of consumed ATP molecules, both per second and per synapse in the primate cortex (using Avogadro number 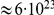 mol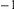, and the fact that about 31 ATP molecules are produces per one used glucose molecule [53]). Thus, the cost of creating and maintaining one synapse in the human cortex during development is about 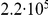 ATP molecules/second, which can increase during adolescence to 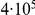 ATP/sec.

There is a growing evidence that a typical excitatory synapse can operate only in a limited number of structurally different discrete states [54], [55]. Since the sizes of synapses (lengths of postsynaptic densities) during postnatal development remain roughly constant [23], [28], one can assume that the number of synaptic states is also approximately invariant. Assuming that a synapse has on average between 10 and 100 states [55], we can estimate the amount of ATP utilization per 1 bit of stored synaptic information. For human brain we obtain 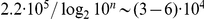 ATP/bit per second, where 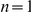 or 2. Thus, during a human lifetime (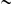
80 years) a typical synapse uses 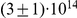 ATP molecules per stored 1 bit of information.

Invariants in the brain design or dynamics are not too numerous, and their existence clearly deserves more attention and thought. The current finding about the constant energy per synapse during development (for a given brain region) expands a short list of the discovered invariants, including adult synaptic density across mammals [56], [57], volume-specific metabolic scaling exponent across gray matter (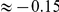) [21], energy per neuron across mammals [58], [59], blood flow and capillary length per neuron [59], or fraction of brain volume taken by glia across mammals [60], [61]. It seems that there are some common principles underlying these invariants, which could be related to the economy of brain wiring [3], [4], [5], [62], [63], [64], [65]. This in turn could be associated with the evolutionary constraints coming from limited energetic resources [19], [20], [31], as the brain is an energy-expensive organ [18], [21], and synapses were pointed out as one of the important users of the cerebral metabolism [21], [31], [32], [33]. The fact that cerebral metabolic rate CMR and synaptic density 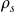 are rather strongly positively correlated (Table 4, Fig. 3) speaks in support of the last argument.

The results in this study indicate that synapses are even bigger energy users than previously estimated. Calculations presented in Tables 1–3 show that at adulthood, when synaptic density is generally lower than in adolescence, synapses can still consume about 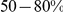 of the total glucose consumption rate. For example, for rat cortex 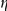 is either 0.48 (visual) or 0.81 (parietal). The average of these values is about twice the amount that was previously calculated for adult rat cortex [31]. The likely source of the discrepancy is the probability of neurotransmitter release, which was calculated here as 0.45 (for rat visual cortex), and assumed in [31] as 0.25. Generally, it should be kept in mind that the computed values of the release probability are only averages, as this parameter is highly variable in time and additionally input specific, and could be somewhere between 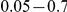 [40], [41], [45], [46]. Because the neurophysiological model of the gray matter metabolism presented in this paper (see Materials and Methods) yields reasonable numerical values of this highly uncertain parameter, it could play a useful role in the future in determining other functional circuit parameters from glucose metabolic data.

It is found that, as a rule, synaptic efficacy (signaling) is negatively correlated with cortical average neural firing rate across all examined species (Fig. 4). Low firing rates usually correspond to high synaptic efficacy, and vice versa (Fig. 4). The interesting feature is that all data points coming from different mammals and cortical regions collapse (with high correlations) into one universal curve given by Eq. (3). This clearly suggests that synaptic regulatory mechanisms such as depression and potentiation are coupled with global network activity and may have a universal cross-species character. This kind of synaptic plasticity is reminiscent of the so-called synaptic scaling, which was found in cortical circuits [66]. In this process, which is typically slow, synaptic efficacy increases if network activity is too low, and it decreases if network activity is too high. This synapse-network activity coupling serves as a tuning mechanism to balance brain spiking activity, which may be important for preventing pathological dynamic states [67].

The collected empirical data in combination with the theoretical metabolic model allow us to determine average firing rates across mammals during development, from the birth to adulthood. These rates are rather low, generally in the range 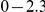 Hz. This probably implies that only a small fraction of cells is active concurrently, which is compatible with an idea of sparse neural coding in cortical networks [19], [31]. Moreover, our results show that larger brains tend to have a slightly lower spiking activities than smaller brains (Table 4). This conclusion that was reached here for developing brains is in line with a previous estimate made for several adult mammals, also using glucose metabolic data [33]. The current interesting finding is that neural firing rate could change during development in coordination with the changes in synaptic density (Table 4). Such dependence improves the goodness of fits for several brain regions significantly.

The semi-empirical results of this study can have some impact on modeling studies related to the connectivity development in the brain. It has been known for a long time that synaptic development is driven to some extent by global spiking activity of neurons [14], [68]. This coupling has also been incorporated in several formal models dealing with synaptogenesis [16], [69], but it often had abstract forms. It seems that the semi-empirical formula derived here (Eq. 3), allows us for a more realistic approach. Alternatively, this formula could be used as a one of the criterions for verification of modeling studies. Similarly, the finding that there exist a (roughly) constant amount of available energy per synapse during development (Fig. 2; Tables 1–3), has not been explored in computational models. Yet, it could have important theoretical implications.

Although, the empirical data in this paper are concerned with normal development, they could also have some relevance for studies dealing with developmental disorders, such as schizophrenia or autism. There are some strong experimental indications that these mental diseases are associated with altered synaptic connectivity [70], [71]. It would be interesting to know whether in these disorders the amount of metabolic energy per synapse during development is also conserved or not? If not, then how large are deviations form a constancy, and whether this measure is somehow correlated with the degree of mental disorder. This perhaps could have some practical applications.

## Materials and Methods

### Developmental data

The ethics statement does not apply to this study. Experimental data for glucose cerebral metabolic rate (CMR) and synaptic density (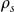) during development for rat, cat, macaque monkey, and human are presented in Tables 1–3. These mammals have adult brains that span 3 orders of magnitude in volume. The metabolic data were collected from the following sources: for rat [34]; for cat [35]; for monkey [36], [37], [38]; for human [30], [39]. The synaptogenesis data were taken from: [22], [23] for rat; [24] for cat; [25], [26], [27], [28] for monkey; and [29] for human.

### Theoretical model of cerebral metabolic rate

In this section we derive an expression for the glucose cerebral metabolic rate CMR in gray matter. This derivation follows closely a detailed analysis presented in [33], and additionally extends it by including also NMDA synaptic currents. We assume that the activities of Na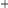/K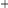 pumps are the major contributors to brain metabolism, which is in agreement with empirical estimates [72], [73]. The main objective of these pumps is to remove Na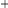 ions from neuron's interior, in order to maintain a negative membrane resting potential, which is critical for all neural functions.

During one cycle, the Na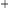/K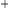 pump extrudes 3 Na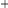 and intrudes 2 K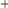 ions, which translates into a net removal of one elementary positive charge that comprises a pump current 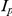. Consequently, the pump current 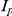 constitutes of only 1/3 of the total sodium current through the membrane. In terms of the metabolic cost, this pumping process uses 1 ATP molecule (per one cycle) to remove one positive charge. The metabolic expenditure of this process in the long run depends on the level of intracellular sodium concentration.

According to biochemical estimates [53], about 31 ATP molecules are made per one oxidized glucose molecule during cellular respiration. Consequently, the glucose metabolic rate CMR (the amount of moles of glucose per tissue volume and time) is given by
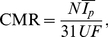
(4)where 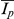 is the average net pump current, 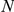 is the number of neurons contained in the gray matter volume 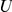, and 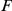 is the Faraday constant. The ratio 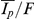 is the amount of moles of ATP molecules consumed on average per neuron per time unit.

At the steady state, i.e. for constant firing rates and after averaging over long times (hundred of seconds to several minutes), the average sodium concentration inside neurons is relatively stable [33]. This corresponds to the situation when the pump current 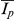 balances 3 different types of sodium currents through the membrane [33]:
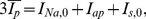
(5)where 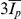 is the amount of Na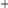 charge per second that is removed by the Na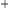/K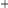 pump. The current 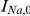 is Na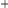 influx through sodium channels at rest (a small contribution), 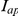 is Na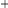 influx due to action potentials, and 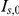 is the sodium influx through synapses during background dendritic synaptic activity. The explicit forms of the first two currents are given by:
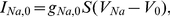
(6)

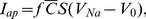
(7)where 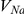 is the reversal potential for Na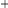 ions, 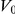 is the resting membrane potential, 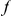 is the average firing rate, 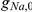 is the resting Na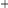 conductance per unit area, 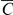 is effective membrane capacitance per unit area, and 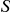 is the neuron's membrane surface area.

The synaptic contribution 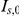 to the sodium influx is proportional to a temporal average over an interspike interval of the AMPA and NMDA synaptic currents, and takes the form:
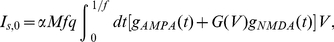
(8)where 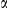 is the proportionality factor between the total synaptic current and Na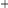 influx current and is given by 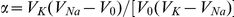, where 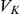 is the reversal potential for K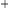 ions. The latter dependence can be easily computed [33] and follows from the fact that AMPA current is composed exclusively of Na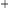 and K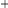 ions, and NMDA current is composed largely of these ions (the influence of Ca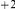 is neglected here, as it constitutes only of about 7–10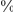 of the NMDA current [74]). The symbol 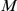 denotes number of synapses per neuron, 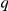 is the neurotransmitter release probability, and 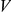 is neuron's membrane voltage. The function 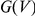 is a voltage-dependent factor associated with NMDA receptors given by [44]: 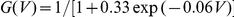, where 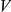 is in mV. For voltage equal to the resting potential, i.e. 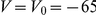 mV, we obtain 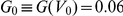. The symbols 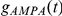 and 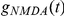 denote the time dependent single synapse conductances, respectively AMPA and NMDA type. Below, we assume that the rising phase of these conductances is much faster than their decaying phases. That is, we take 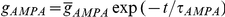, and 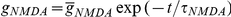, where 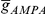, 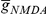 are the peak conductances, and 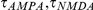 are corresponding decay time constants. Also, since the duration of a single action potential is very short in comparison to the average interspike interval 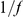, we can assume that for the most time 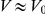 under the integral. With these assumptions we can carry out the integration in Eq. (8), with the result
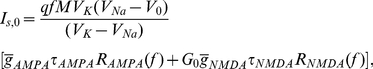
(9)where the frequency dependent factor 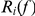 (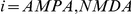) has the form: 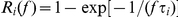. This factor for the AMPA current is practically always close to 1, as 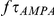 is significantly smaller than unity even for firing rates 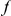 as large as 100 Hz (with 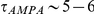 msec). Generally, for the NMDA current 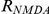 is less than 1, and could be even 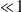 for very large 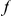. However, for the empirical frequencies found in this study (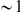 Hz), the factor 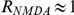. Consequently, the values of 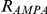 and 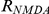 are both taken as 1 further in the analysis.

Combination of Eqs. (4–7) and (9) yields an approximate glucose metabolic rate CMR as follows:
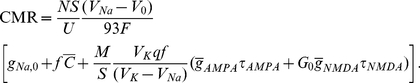
(10)


Additionally, we assume that the geometry of axons and dendrites can be approximated as cylindrical with equal volumes [56]. Thus, we can write the total membrane surface area as 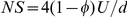, where 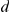 is an effective fiber diameter (harmonic mean of axonal and dendritic diameters), and 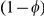 is the fraction of volume taken by neural wiring [33]. Moreover, the surface density of synapses can be written as 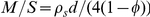, where 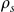 is the synaptic density [33]. Substituting the above expressions for 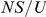 and 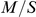 into Eq. (10), we obtain CMR in a more convenient form:
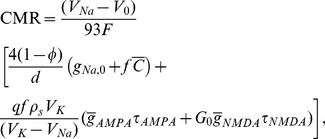
(11)or equivalently with an explicit dependence of CMR on synaptic density and firing rate as:
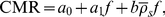
(12)where the coefficients 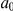, 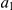, and 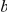 are given by
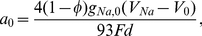
(13)

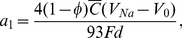
(14)and
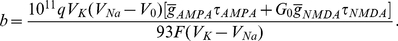
(15)


In Eq. (12) the firing rate 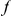 is in Hz, and the symbol 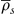 denotes the synaptic density amplitude defined as 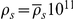, where 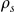 is expressed in cm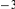. The coefficients 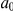 and 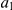 are invariant or nearly invariant across species, and they do not seem to change significantly during development after birth. This is because they depend on the parameters, which themselves are developmentally or species independent. These are electrical voltages (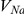, 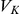, 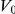) due to their logarithmic dependencies on ionic concentrations, membrane capacity 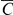, and structural parameters: the fraction of volume taken by wiring 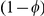 or fraction of neuropil [26], [27], [28], and the effective wire thickness 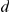 [56]. Also the sodium conductance at neuron's rest is very small, and biophysical models suggest that it is similar across species. The numerical values of these parameters are: 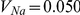 V, 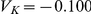 V, 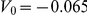 V (standard values), 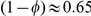 [26], [27], [28], [56], 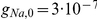 (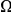
cm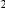)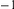 [33], 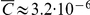 F/cm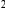, and 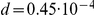 cm [33]. Based on these values, we obtain 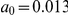 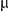
mol/(g

min), and 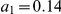 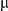
mol

s/(g

min). The parameter 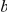 is related to synaptic activities, and its value is determined in the Results section for every species and brain region.

There are no data on *in vivo* firing rates during development. Therefore, we have to assume some form of 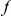. We consider two scenarios for this quantity. In the simplest case, firing rate and synaptic density are independent of each other, and we take 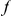 to be a constant. In a second case, we assume that firing rate and synaptic density are correlated in such a way that 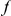 is an increasing function of 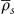. This follows from a simple expectation that higher synaptic density generally mean more excitatory synaptic input to a typical neuron, as 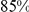 of synapses in the cerebral cortex are excitatory [56], [57]. More excitatory input in a recurrent network translates into higher average firing rates. This is in agreement with mean-field models of recurrent neural networks [75]. Thus, the simplest expression for the firing that combines both scenarios is 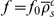, where 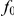 and the exponent 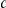 are to be determined by a fitting procedure to the data. When 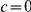, then 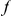 is independent of synaptic density.
